# Supplementary material for: The Drosophila melanogaster enzyme glycerol-3-phosphate dehydrogenase 1 is required for oogenesis, embryonic development, and amino acid homeostasis
Source: G3 (Bethesda). 2022 May 10;12(8):jkac115. doi: 10.1093/g3journal/jkac115 (PMC9339270; doi:10.1093/g3journal/jkac115)
Supplement: jkac115_Supplemental_Material_Legend [file jkac115_supplemental_material_legend.docx]

**SUPPLEMENTAL TABLES**

**Table S1. GC-MS analysis of control, *Gpdh1* zygotic mutants, and *Gpdh1* maternal-zygotic mutants.** Samples contained 25 mid-L2 larvae. Data normalized to sample mass and a d4-succinic acid internal standard.
